# Identification of HER2-over-expression, HER2-low-expression, and HER2-zero-expression statuses in breast cancer based on ^18^F-FDG PET/CT radiomics

**DOI:** 10.1186/s40644-025-00880-2

**Published:** 2025-05-12

**Authors:** Xuefeng Hou, Kun Chen, Huiwen Luo, Wengui Xu, Xiaofeng Li

**Affiliations:** 1 Department of Molecular Imaging and Nuclear Medicine, Tianjin Medical University Cancer Institute and Hospital, National Clinical Research Center for Cancer, Huan-Hu-Xi Road, Ti-Yuan-Bei, He Xi District, Tianjin, 300060 China; 2 Tianjin’s Clinical Research Center for Cancer, Tianjin, 300060 China; 3 Key Laboratory of Breast Cancer Prevention and Therapy, Tianjin Medical University, Ministry of Education, Tianjin, 300060 China; 4 Department of Nuclear Medicine, Hangzhou Institute of Medicine (HIM), Zhejiang Cancer Hospital, Chinese Academy of Sciences, Hangzhou, 310022 Zhejiang China

**Keywords:** PET/CT, Radiomics, Breast cancer, HER2

## Abstract

**Purpose:**

According to the updated classification system, human epidermal growth factor receptor 2 (HER2) expression statuses are divided into the following three groups: HER2-over-expression, HER2-low-expression, and HER2-zero-expression. HER2-negative expression was reclassified into HER2-low-expression and HER2-zero-expression. This study aimed to identify three different HER2 expression statuses for breast cancer (BC) patients using PET/CT radiomics and clinicopathological characteristics.

**Methods and materials:**

A total of 315 BC patients who met the inclusion and exclusion criteria from two institutions were retrospectively included. The patients in institution 1 were divided into the training set and the independent validation set according to the ratio of 7:3, and institution 2 was used as the external validation set. According to the results of pathological examination, all BC patients were divided into HER2-over-expression, HER2-low-expression, and HER2-zero-expression. First, PET/CT radiomic features and clinicopathological features based on each patient were extracted and collected. Second, multiple methods were used to perform feature screening and feature selection. Then, four machine learning classifiers, including logistic regression (LR), k-nearest neighbor (KNN), support vector machine (SVM), and random forest (RF), were constructed to identify HER2-over-expression vs. others, HER2-low-expression vs. others, and HER2-zero-expression vs. others. The receiver operator characteristic (ROC) curve was plotted to measure the model’s predictive power.

**Results:**

According to the feature screening process, 8, 10, and 2 radiomics features and 2 clinicopathological features were finally selected to construct three prediction models (HER2-over-expression vs. others, HER2-low-expression vs. others, and HER2-zero-expression vs. others). For HER2-over-expression vs. others, the RF model outperformed other models with an AUC value of 0.843 (95%CI: 0.774–0.897), 0.785 (95%CI: 0.665–0.877), and 0.788 (95%CI: 0.708–0.868) in the training set, independent validation set, and external validation set. Concerning HER2-low-expression vs. others, the outperformance of the LR model over other models was identified with an AUC value of 0.783 (95%CI: 0.708–0.846), 0.756 (95%CI: 0.634–0.854), and 0.779 (95%CI: 0.698–0.860) in the training set, independent validation set, and external validation set. Whereas, the KNN model was confirmed as the optimal model to distinguish HER2-zero-expression from others, with an AUC value of 0.929 (95%CI: 0.890–0.958), 0.847 (95%CI: 0.764–0.910), and 0.835 (95%CI: 0.762–0.908) in the training set, independent validation set, and external validation set.

**Conclusion:**

Combined PET/CT radiomic models integrating with clinicopathological characteristics are non-invasively predictive of different HER2 statuses of BC patients.

**Supplementary Information:**

The online version contains supplementary material available at 10.1186/s40644-025-00880-2.

## Introduction

Breast cancer is the most common cancer type in female malignancies, which accounts for 30% of incidence and 15% of mortality, respectively [1, 2]. As a prototypic oncogene, human epidermal growth factor receptor 2 (HER2) is an important driver gene, therapeutic target, and prognostic indicator of breast cancer [3]. HER2-over-expression breast cancer has a higher recurrence rate and a poorer prognosis than HER2-negative breast cancer [3, 4]. Nevertheless, according to a recently proposed classification system, HER2 expression statuses were categorized into the following three groups: HER2-over-expression, HER2-low-expression, and HER2-zero-expression [5, 6]. In other words, the previous HER2-negative status was reclassified into HER2-low-expression and HER2-zero-expression. HER2 is currently a promising therapeutic target in BC targeted therapy. A significant percentage of BC patients benefit from anti-HER2 treatment in clinical practice. As previously reported, compared to BC with HER2-zero-expression, BC with HER2-over-expression exhibited a remarkably favorable prognosis in the context of anti-HER2 targeted therapy [7]. For BC with HER2-low-expression, which accounts for 40–50% of all BC patients, the antibody–drug conjugate (ADC) regimen is increasingly considered a promising, precise treatment strategy [8–10]. Therefore, it is critical to accurately identify HER2 expression status to determine appropriate treatment options for breast cancer patients. In particular, the newly introduced HER2-low-expression status enables a treatment revolution in the BC cohort with HER2-low-expression status. Because HER2-low-expression was previously equal to HER2-negative-expression, anti-HER2 targeted therapy was unavailable to BC with HER2-low-expression status. Pathological analysis of biopsy specimens is typically used for preoperative HER2 status determination based on the results of using immunohistochemistry (IHC) assay and fluorescence in situ hybridization (FISH) test [4]. However, biopsy is still an invasive procedure, and preoperative core needle biopsy (CNB) is only obtained with a limited size and is not able to accurately reflect the overall heterogeneity of the entire tumor [11]. Therefore, a precise, practical, and non-invasive technique to preoperatively identify the three different HER2 expression statuses is urgently needed.

[^18^F]-Fluorodeoxyglucose positron emission tomography/computed tomography ([^18^F]FDG PET/CT) imaging, as a hybrid imaging technology, is widely used in clinical practice for tumor diagnosis and treatment based on simultaneous anatomic structure information and molecular function information. About BC, though PET/CT imaging was previously recommended to be performed for only advanced BC patients, the updated National Comprehensive Cancer Network (NCCN) guidelines suggested that PET/CT should be available to BC patients whose staging was uncertain. Furthermore, multiple PET metabolic parameters, such as standardized uptake value (SUV), metabolic tumor volume (MTV), and total lesion glycolysis (TLG), were also identified as potential indicators to classify molecular subtype for BC [12, 13]. Additionally, HER2-targeted positron emission tomography (PET/CT) enables real-time monitoring of HER2 expression in systemic lesions [14]. However, those traditional PET metabolic metrics are all semi-quantitative indices, which are not able to reflect the complex heterogeneity across the whole lesion in BC. Radiomics, as a rapidly emerging technique, is capable of comprehensively quantifying intra-tumoral and inter-tumoral heterogeneities based on a high-throughput extraction of quantitative features from many medical images [15]. As reported in our previous reports, constructed radiomic models based on PET/CT were verified as potential non-invasive tools to effectively predict the molecular subtype classification in BC, particularly for the HER2 expression status [16, 17].

With the updated stratification strategy for HER2 expression status, it is urgently needed to evaluate the role of PET/CT radiomics in the prediction of HER2-over-expression, HER2-low-expression, and HER2-zero-expression, which is rarely reported in previous studies. In the present study, a total of 215 BC patients from our institution were included to construct and validate the predictive power of radiomic models based on PET/CT in identifying HER2 expression status. It is noteworthy that another 100 BC patients from an external institution were also enrolled to perform an external validation.

## Methods

### Patients

**Fig. 1:**
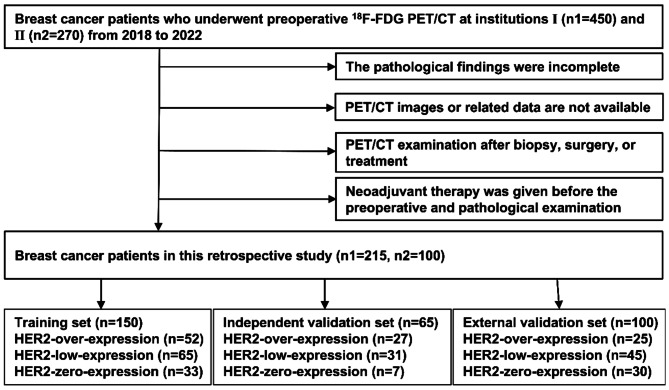
The flow chart of patient enrollment

A total of 315 BC patients who underwent preoperative PET/CT examination were enrolled in this study, 215 from our institution and 100 from another institution. The specific inclusion and exclusion criteria are listed as follows: Inclusion criteria: (1) Patients with pathologically diagnosed primary BC; (2) Preoperative PET/CT examination; (3) Complete and available IHC and/or FISH results for HER2 expression status after surgery. Exclusion criteria: (1) The pathological findings were incomplete; (2) PET/CT images or related data are not available; (3) PET/CT imaging was performed after biopsy, surgery, or treatment; (4) Neoadjuvant therapy was given before the preoperative and pathological examination. The flow chart of patient enrollment is shown in Fig. 1. Patient informed consent was not required for this retrospective investigation, which was permitted by the institutional Ethics Committee.

### Clinicopathological features

Clinical features (Age, SUVmax, SUVmean) and pathological characteristics (estrogen receptor [ER] status, progesterone receptor [PR] status, Ki-67 index) were collected from the hospital system. ER/PR thresholds ≥ 1% and Ki-67 thresholds ≥ 14% are positive.

### Classification of HER2 expression status

The HER2 expression statuses for all the included BC patients in this study were determined by IHC or FISH assay according to the American Society of Clinical Oncology/College of American Pathologists (ASCO/CAP) criteria. Based on IHC and/or FISH results, BC patients were divided into the following three groups: HER2-over-expression (IHC 3 + or 2 + with FISH positive amplification), HER2-low-expression (IHC 1 + or 2 + without FISH positive amplification), and HER2-zero-expression (IHC 0).

### Image acquisition

Institution 1 uses the GE Discovery Elite PET/CT scanner (GE Medical Systems) for PET/CT imaging. Institution 2 performed PET/CT examinations using the Discovery 710 PET/CT (GE Healthcare, Milwaukee, WI, USA). The detailed ^18^F-FDG PET/CT imaging protocol can be found in the Supplementary Material.

### Volume of interest segmentation for breast lesions

The 3D Slicer program (version 5.2.1) and Python (version 3.7.9) were used to perform volume of interest (VOI) segmentation and extract radiomics features. In brief, the VOI in the CT image was delineated layer by layer manually, and the VOI in the PET image was segmented by using a threshold method (40% of the SUVmax) [18]. To guarantee the reliability of VOI segmentation, two experienced nuclear medicine doctors (Readers 1 and 2 have 6 and 11 years of expertise in diagnosing PET/CT images) segmented the lesions on the images, with one of the doctors (Reader 1) performing the segmentation on the images twice.

### Radiomic features extraction

Before radiomic feature extraction, the VOIs in CT and PET images were resampled to 1.0 × 1.0 × 1.0 mm^3^ isotropic voxels [19]. Subsequently, using Pyradiomics, an open-source Python software, a high throughput of radiomic features was extracted based on PET and CT images, respectively. For each included BC patient, a total of 1886 radiomics features were obtained, including 943 PET radiomics features and 943 CT radiomics features. The information for the categories of extracted radiomics features is described in detail in the Supplementary Material. Reliability was determined using intraclass correlation coefficients (ICCs). To calculate the extracted radiomic features’ stability and reliability, inter-(Reader 1 vs. Reader 2) and intra-observer (Reader 1 vs. Reader 1) ICCs were computed. Both inter-(Readers 1 vs. Readers 2) and intra-observer (Readers 1 vs. Readers 1) ICCs > 0.75 were selected in subsequent radiomic analysis.

### Construction and evaluation of radiomic models

**Fig. 2:**
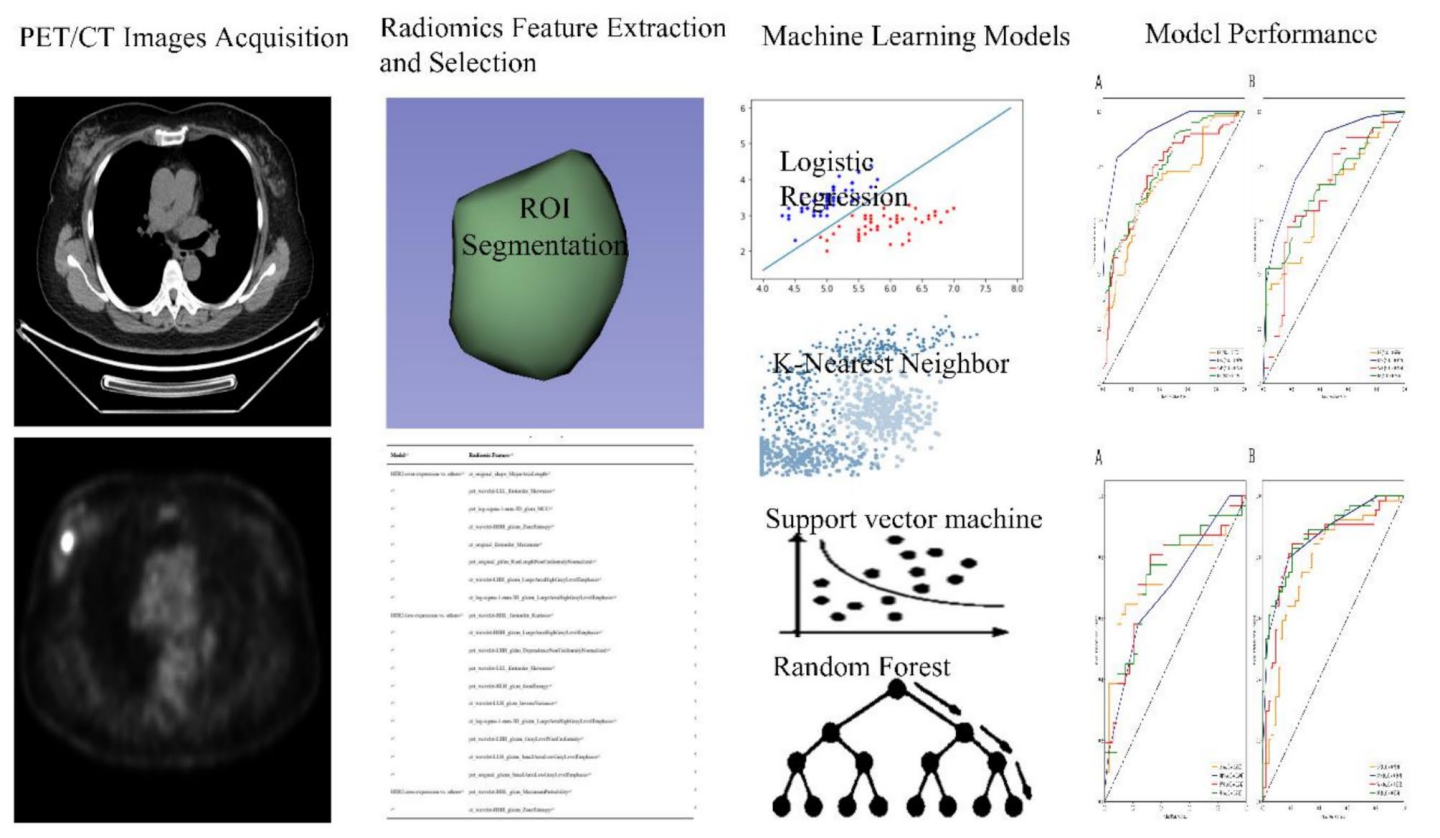
Process of radiomics research

The included BC patients in our institution were used to construct radiomic models and perform internal validation, with the whole population randomly assigned to a training set (*n* = 150) and an independentvalidation set (*n* = 65) at a ratio of 7:3. Based on the HER2 expression status, BC patients were categorized into HER2-over-expression vs. others (HER2-low-expression + HER2-zero-expression), HER2-low-expression vs. others (HER2-over-expression + HER2-zero-expression), and HER2-zero-expression vs. others (HER2-over-expression + HER2-low-expression). According to the above groups, the corresponding radiomics models were constructed to distinguish HER2-over-expression vs. others, HER2-low-expression vs. others, and HER2-zero-expression vs. others. In the training set, T-tests and maximum relevance minimum redundancy (mRMR) were used to screen and finally select the significantly informative radiomic features. Based on that, combined radiomic models were established by integrating clinicopathological features. To select the potentially optimal radiomic models, a total of four machine learning classifiers were employed to establish radiomic models, including logistic regression (LR), k-nearest neighbor (KNN), support vector machine (SVM), and random forest (RF). The receiver operating characteristic (ROC) curves were drawn, and the area under the curve (AUC) values were calculated to assess the predictive performance of the constructed radiomic models. The whole procedure of the radiomic analysis in this study is depicted in Fig. 2.

### Statistical analysis

SPSS (version 26.0), Python (version 3.7.9), and R software (version 4.3.1) were used for all statistical analyses in the study. Regarding clinicopathological features, ANOVA was conducted for continuous variables, whereas Fisher’s exact or Pearson chi-square test was carried out for categorical variables. The Delong test was used to compare the AUCs of different radiomic models.

## Results

### Clinicopathological characteristics in the internal and external institutions

The general clinicopathological characteristics of the included BC patients are presented based on the HER2 expression status in Tables 1 and 2 for the internal and the external institutions, respectively. Particularly, for the internal institution, the included 215 BC patients were randomly divided into a training set and an independent validation set at a ratio of 7:3. As shown in Tables 1 and 2, the differences in the ER and PR statuses are found to be statistically significant between the three groups with different HER2 expression status in the training set (ER: *p* = 0.001; PR: *p* < 0.001), the independent validation set (ER: *p* = 0.036; PR: *p* = 0.001) and the external validation set (ER: *p* < 0.001; PR: *p* < 0.001). Thus, the ER and the PR statuses were selected as the significant clinicopathological features and finally incorporated into the subsequently constructed radiomic models.

**Table 1:** Clinicopathological characteristics of BC patients enrolled in internal institutions

Clinicopathologic Characteristic	Training set (*n* = 150)					Independent validation set (*n* = 65)			
	HER2-over (*n* = 52)	HER2-low (*n* = 65)	HER2-zero (*n* = 33)	*p*		HER2-over (*n* = 27)	HER2-low (*n* = 31)	HER2-zero (*n* = 7)	*p*
Age	50.79 ± 11.05	52.8 ± 12.46	52.36 ± 13.31	0.660		54.00 ± 10.62	53.71 ± 11.69	60.57 ± 13.0	0.341
SUVmax	11.41 ± 5.67	10.96 ± 6.30	11.14 ± 5.76	0.921		11.91 ± 4.98	10.16 ± 4.90	10.83 ± 6.74	0.436
SUVmean	6.55 ± 3.35	6.60 ± 3.72	6.66 ± 3.91	0.983		7.10 ± 2.83	6.12 ± 2.84	6.12 ± 3.81	0.425
ER				0.001					0.036
Positive	23(15.3%)	51(34.0%)	22(14.7%)			16(24.6%)	26(40.0%)	3(4.6%)	
Negative	29(19.3%)	14(9.3%)	11(7.3%)			11(16.9%)	5(7.7%)	4(6.2%)	
PR				< 0.001					0.001
Positive	14(9.3%)	45(30.0%)	18(12.0%)			19(29.2%)	23(35.4%)	2(3.1%)	
Negative	38(25.3%)	20(13.3%)	15(10.0%)			8(12.3%)	8(12.3%)	5(7.7%)	
Ki-67				0.058					0.731
≥ 14	50(33.3%)	65(43.3%)	30(20.0%)			26(40.0%)	29(44.6%)	7(10.8%)	
< 14	2(1.3%)	0(0%)	3(2.0%)			1(1.5%)	2(3.1%)	0(0%)	

Abbreviations: ER, estrogen receptor; PR, progesterone receptor; HER2, human epidermal growth factor receptor 2; SUV: standardized uptake value

**Table 2:** Clinicopathological characteristics of BC patients enrolled in the external institution

Clinicopathologic Characteristic	External validation set (*n* = 100)			
	HER2-over (*n* = 25)	HER2-low (*n* = 45)	HER2-zero (*n* = 30)	*p*
Age	52.16 ± 8.89	54.98 ± 10.36	46.13 ± 10.81	0.002
SUVmax	13.15 ± 5.94	11.43 ± 7.62	13.67 ± 6.52	0.344
SUVmean	6.05 ± 1.68	4.97 ± 1.86	5.85 ± 1.60	0.025
ER				< 0.001
Positive	14(14.0%)	34(34.0%)	17(17.0%)	
Negative	11(11.0%)	11(11.0%)	13(13.0%)	
PR				< 0.001
Positive	11(11.0%)	29(29.0%)	15(15.0%)	
Negative	14(14.0%)	16(16.0%)	15(15.0%)	
Ki-67				0.064
≥ 14	25(25.0%)	40(40.0%)	28(28.0%)	
< 14	0(0%)	5(5.0%)	2(2.0%)	

Abbreviations: ER, estrogen receptor; PR, progesterone receptor; HER2, human epidermal growth factor receptor 2; SUV: standardized uptake value

### Construction of PET/CT-derived radiomic models

Following the aforementioned traditional radiomic analysis procedure, including VOI delineation, radiomic feature extraction, and radiomic feature screening, a total of 8, 10, and 2 radiomic features were finally selected as the informative indicators to discriminate between HER2-over-expression vs. others, HER2-low-expression vs. others, and HER2-zero-expression vs. others, respectively. All the selected radiomic features to identify HER2 expression status are listed in detail in Table 3. In the end, based on a combination of these selected radiomic features with the selected clinicopathological features (ER and PR), four machine learning classifiers were employed, including LR, KNN, SVM, and RF, to develop and select the optimal radiomic models to distinguish HER2-over-expression vs. others, HER2-low-expression vs. others, and HER2-zero-expression vs. others, respectively.

**Table 3:** Final selected radiomics features to identify HER2 expression status

Model	Radiomic Feature
HER2-over-expression vs. others	ct_original_shape_MajorAxisLength
	pet_wavelet-LLL_firstorder_Skewness
	pet_log-sigma-1-mm-3D_glcm_MCC
	ct_wavelet-HHH_glszm_ZoneEntropy
	ct_original_firstorder_Maximum
	pet_original_glrlm_RunLengthNonUniformityNormalized
	ct_wavelet-LHH_glszm_LargeAreaHighGrayLevelEmphasis
	ct_log-sigma-1-mm-3D_glszm_LargeAreaHighGrayLevelEmphasis
HER2-low-expression vs. others	pet_wavelet-HHL_firstorder_Kurtosis
	ct_wavelet-HHH_glszm_LargeAreaHighGrayLevelEmphasis
	pet_wavelet-LHH_gldm_DependenceNonUniformityNormalized
	pet_wavelet-LLL_firstorder_Skewness
	pet_wavelet-HLH_glcm_JointEnergy
	ct_wavelet-LLH_glcm_InverseVariance
	ct_log-sigma-1-mm-3D_glszm_LargeAreaHighGrayLevelEmphasis
	pet_wavelet-LHH_glszm_GrayLevelNonUniformity
	ct_wavelet-LLH_glszm_SmallAreaLowGrayLevelEmphasis
	pet_original_glszm_SmallAreaLowGrayLevelEmphasis
HER2-zero-expression vs. others	pet_wavelet-HHL_glcm_MaximumProbability
	ct_wavelet-HHH_glszm_ZoneEntropy

### Predictive performance of established PET/CT-derived radiomic models in HER2 expression status

**Fig. 3:**
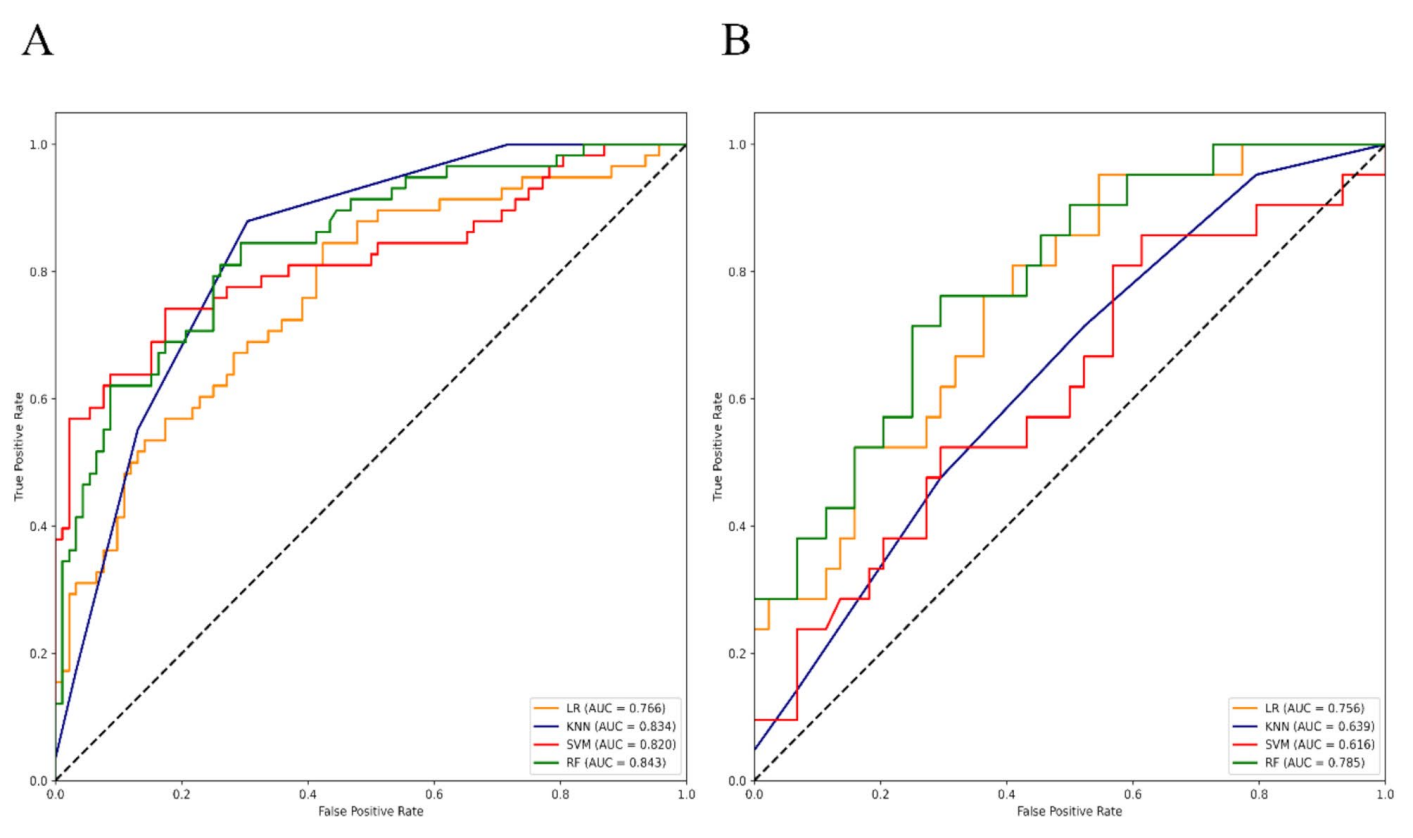
ROC curves of 4 constructed radiomic models for HER2-over-expression vs. others (HER2-low-expression + HER2-zero-expression) in the training (left) and independent validation set (right)

**Fig. 4:**
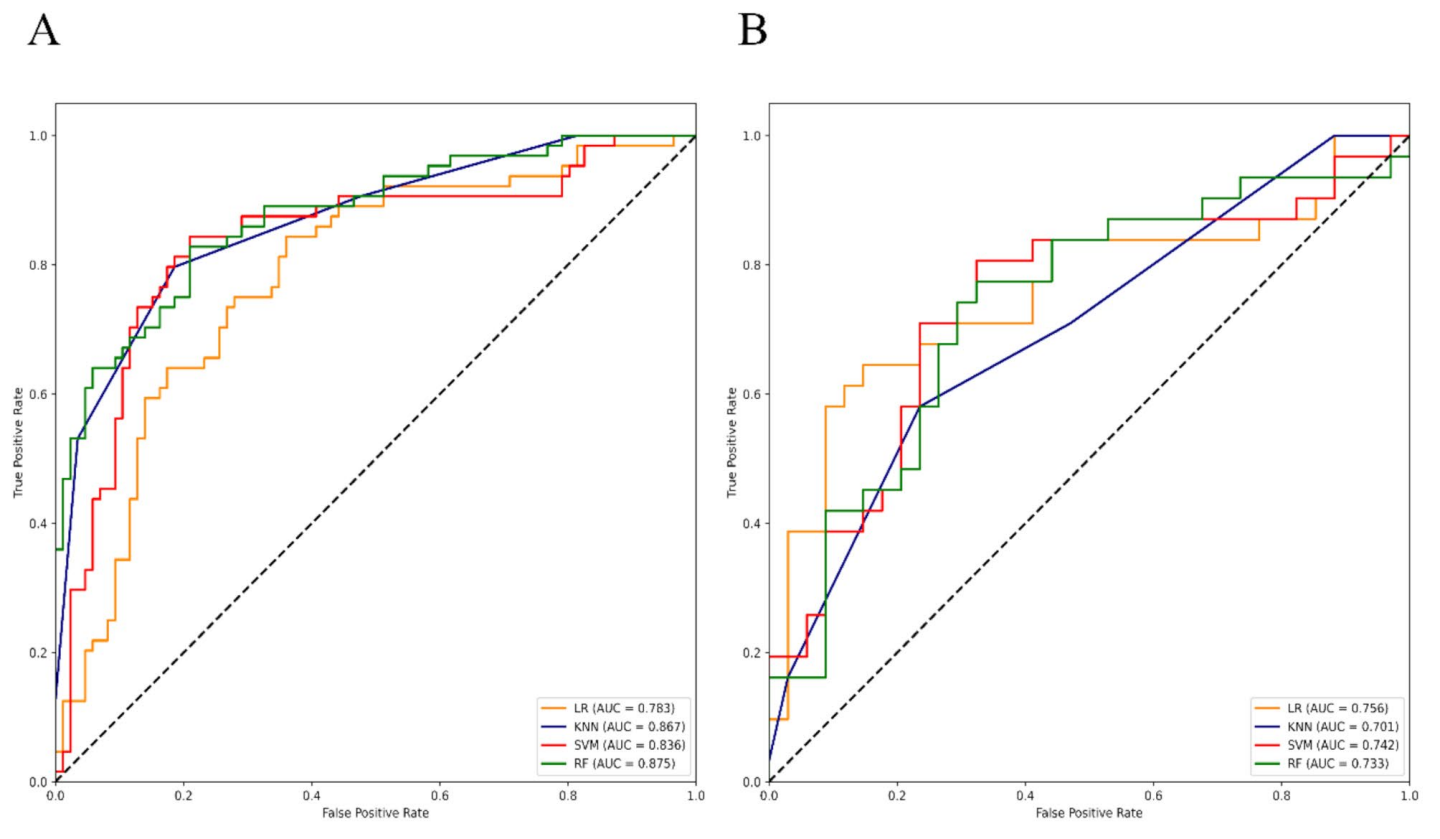
ROC curves of 4 constructed radiomic models for HER2-low-expression vs. others (HER2-over-expression + HER2-zero-expression) in the training (left) and independent validation set (right)

**Fig. 5:**
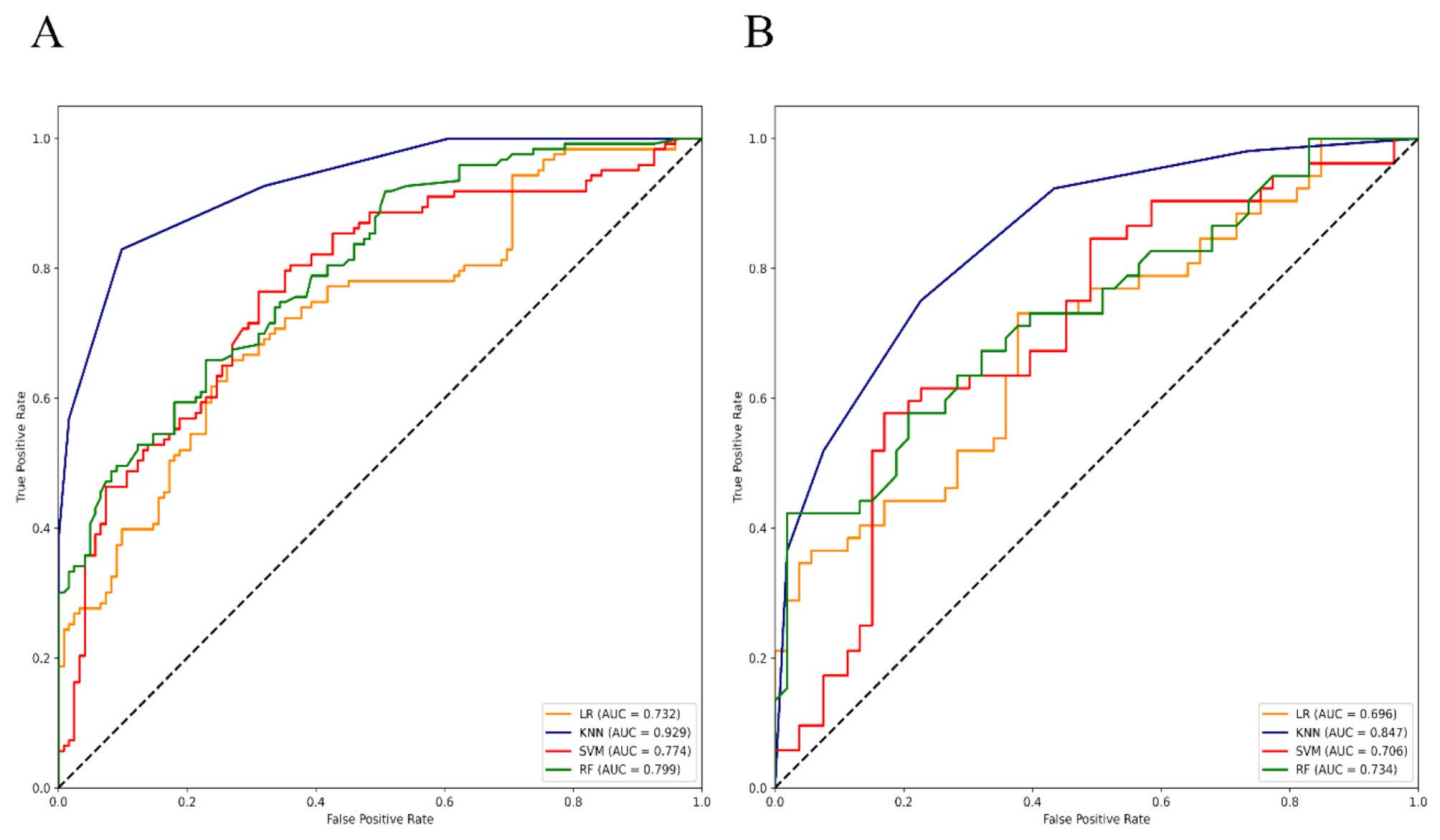
ROC curves of 4 constructed radiomic models for HER2-zero-expression vs. others (HER2-over-expression + HER2-low-expression) in the training (left) and independent validation set (right)

**Fig. 6:**
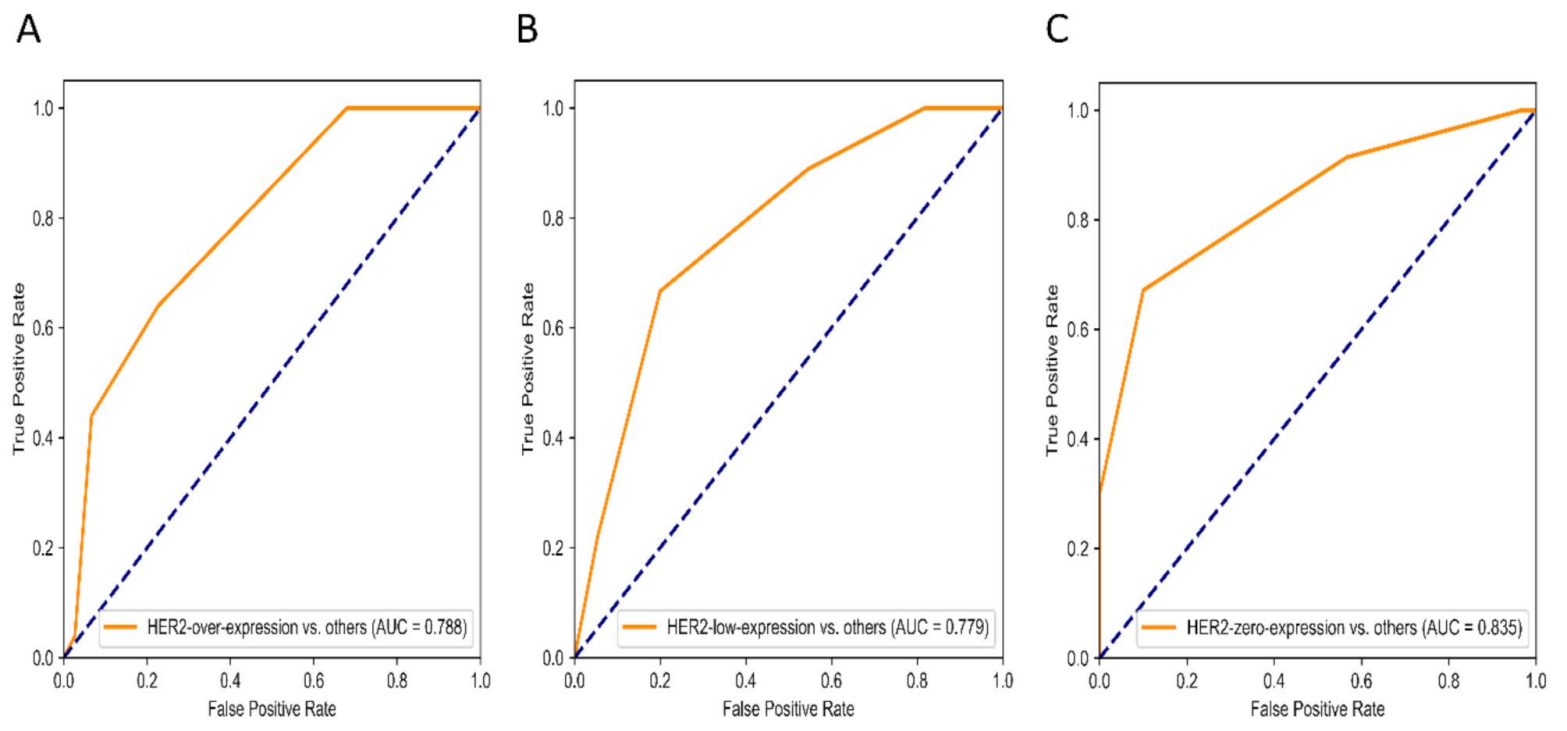
ROC curves of the optimal radiomic models for HER2-over-expression vs. others, HER2-low-expression vs. others, and HER2-zero-expression vs. others in the external independent set

To evaluate the performance of these constructed PET/CT-derived radiomic models in predicting HER2 expression status, respective ROC curves were depicted to calculate AUCs (Figs. 3, 4 and 5, and 6 ). As shown (Table 4), for HER2-over-expression vs. others, the RF model outperformed other models with an AUC value of 0.843 (95%CI: 0.774–0.897) and 0.785 (95%CI: 0.665–0.877) in the training set and the independent validation set, respectively. The DeLong test revealed that the independent validation set’s RF vs. LR (*p* = 0.400), KNN (*p* = 0.025), and SVM (*p* = 0.010) displayed statistical significance. About HER2-low-expression vs. others, the outperformance of the LR model over other models was identified with an AUC value of 0.783 (95%CI: 0.708–0.846) and 0.756 (95%CI: 0.634–0.854) in the training set and the independent validation set, respectively. The DeLong test revealed that the independent validation set’s LR vs. RF (*p* = 0.055), KNN (*p* = 0.012), and SVM (*p* = 0.060) showed statistical significance. Whereas, the KNN model was confirmed as the optimal model to distinguish HER2-zero-expression vs. others, with an AUC value of 0.929 (95%CI: 0.890–0.958) and 0.847 (95%CI: 0.764–0.910) in the training set and independent validation set, respectively. The DeLong test revealed that the independent validation set’s KNN vs. RF (*p* = 0.068), LR (*p* = 0.020), and SVM (*p* = 0.025). Finally, an independent external validation was performed to verify the predictive power of the identified optimal radiomic models in HER2 expression status. As illustrated in Fig. 6, the selected RF model for HER2-over-expression vs. others, the LR model for HER2-low-expression vs. others, and the KNN model for HER2-zero-expression vs. others based on the internal institution, also exhibited predictive performance for BC patients from the external institution with an AUC of 0.788 (95%CI: 0.708–0.868), 0.779 (95%CI: 0.698–0.860), 0.835 (95%CI: 0.762–0.908), respectively.

**Table 4:** The performances of all 12 constructed radiomic models in discriminating HER2 expression status in the training and independent validation sets from the internal institution

Models AUC (95%CI) and datasets	LR	KNN	SVM	RF
HER2-over-expression vs. others				
Training set	0.766(0.690–0.831)	0.834 (0.765–0.890)	0.820(0.749–0.878)	0.843(0.774–0.897)
Independent validation set	0.756(0.634–0.854)	0.639 (0.510–0.755)	0.616(0.488–0.735)	0.785(0.665–0.877)
HER2-low-expression vs. others				
Training set	0.783(0.708–0.846)	0.867(0.802–0.917)	0.836(0.767–0.892)	0.875(0.811–0.923)
Independent validation set	0.756(0.634–0.854)	0.701(0.574–0.808)	0.742(0.618–0.843)	0.733(0.609–0.835)
HER2-zero-expression vs. others				
Training set	0.732(0.672–0.786)	0.929(0.890–0.958)	0.774(0.716–0.825)	0.799(0.744–0.848)
Independent validation set	0.696(0.598–0.782)	0.847(0.764–0.910)	0.706(0.610–0.791)	0.734(0.639–0.815)

## Discussion

The HER2 expression status of BC patients was reclassified as HER2-over-expression, HER2-low-expression, and HER2-zero-expression [20]. Furthermore, previous studies also confirmed that BC patients with HER2-low-expression status and HER2-zero-expression status were different from the perspectives of biological characteristics and responses to treatment [21]. Accordingly, a precise treatment strategy is recommended for BC patients based on HER2 expression status. Traditional HER2-targeted medications significantly improved the prognosis of BC patients with HER2-over-expression status [22, 23], and BC with HER2-zero-expression status is ineligible for HER2-targeted therapy. Whereas, BC with HER2-low-expression status who were previously classified into the HER2-negative expression group would benefit from novel HER2-targeted antibody-drug conjugate (ADC), such as trastuzumab deruxtecan (T-DXd) [4, 24]. For the precision treatment of HER2 BC patients, non-invasive and reliable identification of HER2 expression status before surgery is critically important. In this study, a comprehensive radiomics study based on ^18^F-FDG PET/CT images was performed to construct and select the best radiomics model to predict HER2 expression status. As demonstrated in the results, the predictive performances of different machine learning classifiers varied among different comparison tasks. For HER2-over-expression vs. others, the RF model outperformed other models with an AUC value of 0.785 in the independent validation set. About HER2-low-expression vs. others, the outperformance of the LR model over other models with an AUC value of 0.756 in the independent validation set. Whereas, the KNN model was confirmed as the optimal model to distinguish HER2-zero-expression from others, with an AUC value of 0.847 in the independent validation set. An independent external validation was also conducted in this study. In the external validation set, radiomics models achieved an AUC of 0.788 in differentiating HER2-overexpression from others; 0.779 in differentiating HER2-low-expression from others; and 0.835 in differentiating HER2-zero-expression from others, respectively. The research results show that the radiomics model has potential clinical application value in guiding the treatment of BC patients with different HER2 expression status.

For HER2-over-expression vs. others in the study, which is equal to HER2-over-expression vs. HER2-negative-expression, multiple radiomic models based on PET/CT images were already constructed and evaluated in previous studies. Chen et al. included 217 BC patients and finally constructed four radiomic models, and the XGBoost model outperformed other models in discriminating between HER2-over-expression and HER2-negative with an AUC of 0.72 in the test set [16]. Liu et al. built a radiomic signature model to distinguish between HER2-over-expression and HER2-negative, and the AUC of the model was 0.788 in the validation cohort [17]. In our work, the optimal RF model (Independent validation set: AUC = 0.785, External validation set: 0.788) could distinguish HER2-over-expression from others and had comparable predictive ability to previous studies.

As aforementioned, it is necessary to discriminate HER2-low-expression and HER2-zero-expression, because they are characterized by different biological features, response to anti-HER2 treatment, and different prognosis [21]. Currently, studies to differentiate between HER2-low-expression and HER2-zero-expression status were mainly limited to MRI. Guo et al. established a deep learning radiomic (DLR) model based on magnetic resonance imaging (MRI) to exclusively discriminate between HER2-low-expression and HER2-zero-expression with an AUC of 0.750 [25]. However, the DLR model can only be used to classify HER2-negative status. A study from Ramtohul et al. [26], an MRI-based radiomic model was constructed to distinguish HER2-zero-expression vs. others (HER2-low-expression + HER2-over-expression) with an AUC of 0.80, whereas no predictive radiomic models were developed to differentiate HER2-over-expresssion vs. others, and HER2-low-expression vs. others. In our study, three comparison tasks were completed, including HER2-over-expression vs. others, HER2-low-expression vs. others, and HER2-zero-expression vs. others. Similarly, multiple MRI diffusion models were constructed by Mao et al. to conduct these three comparison tasks [27], but no radiomic or deep learning analysis was used in the study. Though both studies from Zheng et al. [7] and Dai et al. [28] constructed three models for identifying HER2 status based on multi-parameter MRI radiomics and deep learning to complete these three comparison tasks, no clinicopathological characteristics or multiple machine learning classifiers were included to conduct a comprehensive radiomic analysis. To the best of our knowledge, there are currently few radiomics analyses based on PET/CT images to identify the three different HER2 expression statuses of BC. In our study, we performed a comprehensive radiomic analysis based on PET/CT images, including clinicopathological features and multiple machine learning classifiers, to select the optimal radiomic model, which further enhanced the identification of the three HER2 expression statuses.

ER and PR are present in mammary epithelial cells, but they may be partially or completely absent when the normal cells turn into cancerous cells. Multiple mechanisms were potentially responsible for the loss of hormone receptors in BC cells [29]. Both ER and PR are remarkably correlated with HER2 expression status. As reported, HER2-over-expression was negatively correlated with PR or ER level [30]. Patients with HER2-over-expression status had lower ER/PR levels compared to those with HER2 low-expression status [31]. Presumably, that partially contributed to the differences in ER and PR levels between BC with different HER2 expression statuses.

Despite encouraging conclusions obtained in the study, some limitations need to be pointed out. First, manual VOI segmentation on CT images is still a tedious and subjective process. In future studies, standardized segmentation algorithms should be employed to guarantee objectivity and repeatability. Secondly, only two institutions were enrolled to perform internal and external validation. More external institutions were expected to further verify the conclusion in a multi-center study in the future. Then, only BC with invasive ductal carcinoma was included in the study, and no analysis was conducted for other histological types.

## Conclusions

Our study first shows that three different HER2 expression statuses can be identified based on PET/CT radiomic models. Identifying the HER2-over-expression statuses will benefit patients who are undergoing traditional HER2-targeting therapy. Identifying the HER2-low-expression statuses will benefit patients from novel anti-HER2 treatment options (T-DXd).

## Electronic supplementary material

Below is the link to the electronic supplementary material.

Supplementary Material 1

## Data Availability

No datasets were generated or analysed during the current study.
