# Supplementary material for: Identification of HER2-over-expression, HER2-low-expression, and HER2-zero-expression statuses in breast cancer based on 18F-FDG PET/CT radiomics
Source: Cancer Imaging. 2025 May 12;25:62. doi: 10.1186/s40644-025-00880-2 (PMC12070556; doi:10.1186/s40644-025-00880-2)
Supplement: Supplementary file 1 — Supplementary Material 1 [file 40644_2025_880_MOESM1_ESM.docx]

**Supplementary Material**

Identification of HER2-over-expression, HER2-low-expression, and HER2-zero-expression statuses in breast cancer based on ^18^F-FDG PET/CT radiomics

**Image acquisition**

^18^F-FDG PET/CT imaging was performed using a PET/CT scanner for each enrolled BC patient. Before scanning, patients fasted for at least 6 hours, and their blood glucose levels were controlled to <11.1 mmol/L. Sixty minutes after the injection of ^18^F-FDG (3.7-5.55 MBq/kg) into the cubital vein, PET/CT scans were performed from the skull base to the distal femur. For institution 1, PET/CT imaging was conducted using the GE Discovery Elite PET/CT scanner (GE Medical Systems). Initially, a low-dose CT scan was first performed for attenuation correction, and the tube current was 50-80 mAs, the slice thickness was 5 mm, and the tube voltage was 120 kV for the CT scan. Then, the PET was scanned in eight-bed positions, each bed received a two-minute scan, and the increment was 16.2cm (3D mode). PET images were reconstructed using the ordered-subset expectation maximization (OSEM) iterative algorithm, and the final PET voxel size was 5. 3 mm×5. 3 mm×2. 5 mm. For institution 2, PET/CT examination was conducted using the Discovery 710 PET/CT (GE Healthcare, Milwaukee, WI, USA). CT images were obtained for lesion localization (30‑80 mAs, 120 kVp, 5‑mm wide section). PET images were acquired after CT scans (3D mode), encompassing 6‑7 bed positions. PET/CT images were reconstructed using an iterative algorithm. All PET/CT images were independently read by two experienced PET/CT experts, and disagreements were resolved by consensus.

**Radiomic features extraction**

PET and CT images were pre-processed with wavelet filters and the Laplacian of Gaussian (LoG). After preprocessing of PET/CT images, features were extracted from ROIs within PET and CT images using Pyradiomics, an open-source Python package compliant with the image biomarker standardization initiative (IBSI) standard. Following are the several categories of extracted features: (1) first-order features, such as energy, standard deviation, variance, and entropy, which were used to reflect the basic properties of the image; (2) shape features, such as voxel volume, sphericity, and perimeter, which were used to describe the geometry and size properties of the ROI; (3) texture features, such as gray level dependence matrix (GLDM), gray level co-occurrence matrix (GLCM), and gray level run length matrix (GLRLM), which were employed to quantify the heterogeneity of the tumor.
